# Synthesis of legonmycins A and B, C(7a)-hydroxylated bacterial pyrrolizidines

**DOI:** 10.3762/bjoc.17.31

**Published:** 2021-02-02

**Authors:** Wilfred J M Lewis, David M Shaw, Jeremy Robertson

**Affiliations:** 1 Department of Chemistry, University of Oxford, Chemistry Research Laboratory, Mansfield Road, Oxford, OX1 3TA, United Kingdom; 2 Vertex Pharmaceuticals (Europe) Ltd., 86–88 Jubilee Avenue, Milton Park, Abingdon, OX14 4RW, United Kingdom; 3 current address: MSD UK Discovery Centre, Francis Crick Institute, 1 Midland Road, London, NW1 1AT, United Kingdom

**Keywords:** acyloxypyrroles, bacterial pyrrolizidines, cyanoketones, legonmycin, vinylogous ureas

## Abstract

A one-flask, two-step procedure from 3-amino-2-methyl-5,6,7,7a-tetrahydro-1*H*-pyrrolizin-1-one affords the *Streptomyces* secondary metabolites legonmycins A and B – three operations overall from methyl *N*-Boc-prolinate. The key step proceeds in each case via *N*,*O*-diacylation, then selective oxidative hydrolysis of the intermediate bicyclic pyrrole and establishes a precedent for the synthesis of related C(7a)-hydroxylated pyrrolizidines.

## Introduction

**Figure 1:**
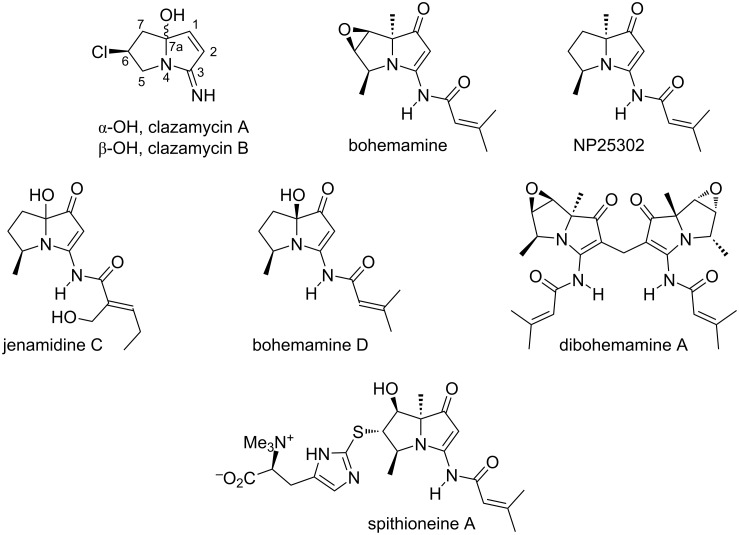
The clazamycins, and selected bacterial pyrrolizidines of the vinylogous urea type. For consistency, the standard numbering convention used for the plant pyrrolizidines, as depicted for the clazamycins, is used throughout.

At least 40 members of the large class of pyrrolizidine alkaloids [1–4] have so far been characterized from bacterial cultures. Of these ‘bacterial pyrrolizidines’, those of the vinylogous urea type are particularly intriguing from a chemical and biosynthetic perspective. Their emergence dates back to 1977 with a patent filing from The Upjohn Company describing an unidentified ‘antibiotic 354’ isolated as a fermentation product of *Streptomyces puniceus* subsp. *doliceus*; spectroscopic characterization, including ^1^H NMR data, was provided, but no structure [5]. In 1980, the same group reported that antibiotic 354 is equivalent to clazamycin B (Figure 1) [6], one of two 7a-hydroxylated iminopyrrolizinones that had been described a year earlier by a group at The Institute of Microbial Chemistry in Tokyo [7–8]. The two clazamycins, A and B, are related as 7a-epimers that interconvert in aqueous solution to form a pH-dependent equilibrium ratio of the two [9]; they are weakly antibacterial and were shown to prolong “the survival period of mice inoculated with leukaemia L-120 cells” [7–8]. Although the clazamycins are not vinylogous ureas, since they lack the C(1) carbonyl group, their structural relationship with bohemamine, the first genuine member of the group, qualifies them as honorary members. The structure of bohemamine, named by its discoverers at Bristol–Myers Company and Cornell University after the opera *La Bohème*, was also reported in 1980 [10]. Following a 23-year hiatus, two papers submitted within two weeks of each other reported, respectively: (1) the isolation from *Streptomyces* sp. UMA-044 and characterization of NP25302, that differs from bohemamine in lacking the 6,7-epoxide functionality [11] and (2) the jenamidines A–C, from another *Streptomyces* sp. strain [12], although these molecules were not recognized as pyrrolizidines until Snider’s subsequent synthetic work revised the structural assignment [13–14] and, separately, established the absolute configuration of natural (+)-NP25302 [15]. Contemporaneously, three further bohemamines were described [16]; five more plus a related 7a-hydroxylated variant [17], various dimeric bohemamines [18–19], and spithioneines A and B [20] extended the range more recently [21].

**Figure 2:**
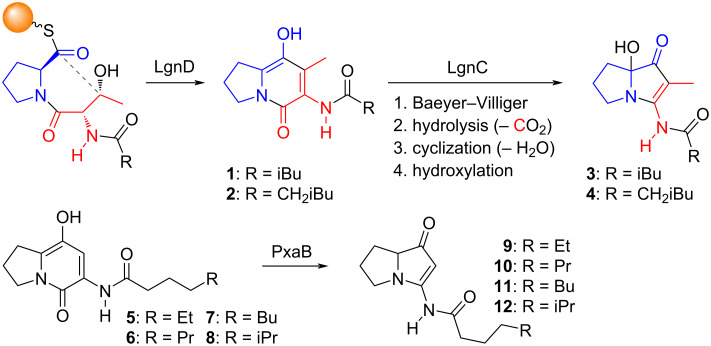
Key species in the biosynthesis of legonmycins A (**3**) and B (**4**), and the pyrrolizixenamides A–D (**9**–**12**).

Almost 40 years after the bacterial pyrrolizidines were first recognized, two 2015 papers addressed their biosynthesis. Thus, the investigation of the metabolites of *Streptomyces* sp. MA37 (from a soil sample obtained in Legon, Ghana) revealed the production of legonmycins A (**3**) and B (**4**) (Figure 2) and found that just four genes (*lgnA–D*) were necessary for their biosynthesis [22]. Proteins coded by three of these genes (LgnA, LgnB, and LgnD) were shown to assemble legonindolizidines A (**1**) and B (**2**) – from proline, threonine, and a fatty acid component – which are then converted by LgnC, a flavin-dependent monooxygenase, into the corresponding legonmycins (**3** and **4**) via a sequence of Baeyer–Villiger-type ring expansion, hydrolysis and decarboxylation, cyclization and dehydration, and finally hydroxylation at C(7a). Just one month later, Bode reported the identification of an unknown gene cluster in the symbiotic bacterium *Xenorhabdus stockiae* [23]. Cloning and expression of this *pxaAB* gene cluster in *E. coli*, and analysis of the metabolites by differential 2D NMR spectroscopy, led to the isolation and characterization of pyrrolizixenamide A (**9**) and, subsequently, pyrrolizixenamides B–D (**10**–**12**). Ultimately, an analogous biosynthetic pathway to that proposed for the legonmycins was established with, in this case, PxaB achieving the oxidative steps from indolizidine intermediates **5**–**8** produced by PxaA. An important aspect of this work was the finding that at least 90 different bacterial strains, spanning 23 species, contain gene sequences encoding proteins related to PxaB. These species include both Gram-positive and Gram-negative bacteria, indicating that pyrrolizidines are potential secondary metabolites of a variety of bacterial genera. The most recent addition to the bacterial pyrrolizidine literature also concerns their biosynthesis and addresses the origin of the methyl groups in the bohemamines and NP25302 [24].

Our interest in this area originated in reports of significant biological activity for those bacterial pyrrolizidines for which such effects have been assessed. The clazamycins, jenamidine A, dibohemamines D–F, and quinohemamine are all reported to be cytotoxic against a variety of cancer cell lines, and both bohemamine and NP25302 inhibit HL-60 cell adhesion to Chinese hamster ovary cells expressing intercellular adhesion molecule ICAM-1 (CD-54). This interest led us to develop a total synthesis of NP25302 [25] and its 5-normethyl analog and, as described in this paper, concise syntheses of the legonmycins.

## Results and Discussion

The legonmycins are unique among the bacterial pyrrolizidines in bearing a C(2) methyl group. The biosynthetic study [22] demonstrates that this methyl group originates from the terminal carbon in threonine and not, as might be thought to be theoretically possible [18–19], via a late-stage addition to an intact pyrrolizidine core. The molecules are isolated as their racemates and, by analogy to the clazamycins, even if the LgnC-mediated hydroxylation is fully stereoselective, the C(7a) center is expected to be configurationally unstable in aqueous solution. Chiral HPLC analysis of samples of pyrrolizixenamide A isolated from culture clearly indicated racemic specimens but also revealed the presence of the two C(7a)-hydroxylated derivatives whose presence in the mixture became enriched during purification. Taken together, it seems reasonable to envisage that ready equilibration of the pyrrolizixenamide enantiomers (and those of related molecules such as jenamidine A and the prelegonmycins) via the hydroxypyrrole tautomer, and aerial oxidation of this electron-rich intermediate, would offer a non-enzymatic route to the various C(7a)-hydroxylated bacterial pyrrolizidines including the legonmycins, jenamidines B and C, and bohemamine D (cf. [26–30]).

With all this in mind, we set out to develop a simplification of Snider’s route [14–15] to the vinylogous urea core for application to the synthesis of legonmycins A and B, expecting to facilitate the C(7a) hydroxylation by exploiting the tendency to C(1–7a) enolization. Since Snider was unsuccessful in adapting his route to encompass the synthesis of jenamidines B and C [15], a successful route to the legonmycins would establish conditions for the synthesis of further C(7a)-hydroxylated bacterial pyrrolizidines and related molecules.

**Scheme 1:**
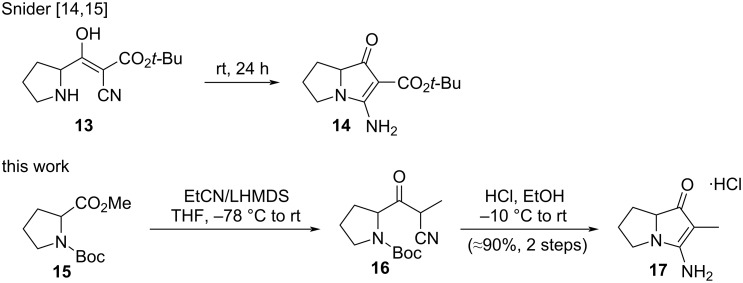
Preparation of the legonmycin core.

Pyrrolizidine **14** (Scheme 1), the key intermediate in Snider’s improved route to jenamidine A and Bode’s preparation of pyrrolizixenamides A (**9**) and D (**12**), is formed by *N-*cyclization onto the nitrile group in cyano-β-ketoester **13**. The appended ester functionality in **14** has, at some point, to be removed, as its presence complicates a potential application to the (2-methyl-substituted) legonmycins, and it exerts a potentially stabilizing electronic effect on pyrrolic intermediates that could be unhelpful in downstream oxidation chemistry. Accordingly, our route began with the addition [31–33] of 2-lithiopropionitrile to methyl *N-*Boc-prolinate (**15**) to give α-cyanoketone **16** which was used in crude form or purified by chromatography to a colorless solid, albeit with significant loss in yield. The removal of the Boc protecting group under acidic conditions was accompanied by cyclization in situ [14–15,34–35] and pyrrolizinone derivative **17** was obtained efficiently on a multigram scale over two steps.

**Scheme 2:**
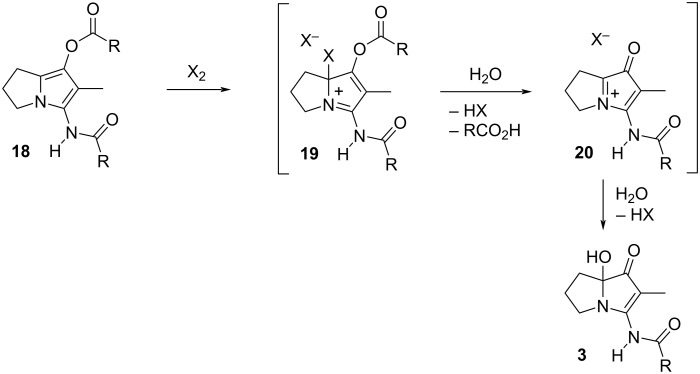
Hypothesis for the oxidative hydrolysis of diacylated pyrrolizidinone **18** (R = iBu).

Originally, it was expected that adapting the conditions (NaH, RCOCl, THF) used for the acylation step in the synthesis of NP25302 [15,25] would deliver both prelegonmycins by the appropriate choice of the acylating agent. In the event, no monoacylated product was observed and attempts to isolate the individual components from the crude mixtures by chromatography provided material of insufficient purity to allow the definitive assignment of the structures. Diacylation is a feature of reactions of this type, with both Snider and Bode observing such side-products, although the extent of diacylation in those cases appears to be reduced, perhaps by virtue of the 2-carboxyl group present in their work. Recognizing that the ester and amide carbonyl groups in the diacylation product (**18**, Scheme 2) should be easily differentiated, and that the ester represents an enol(ate) equivalent, improved conditions for the diacylation were developed. Ultimately, the reaction with isovaleryl chloride in acetonitrile, with pyridine as a scavenger for the liberated HCl, gave solutions of crude diacylated product **18** in acceptable purity after simple filtration as work-up. It was reasoned that the activation of the electron-rich pyrrole (with generic electrophile X_2_ as shown) would hasten cleavage of the ester and that, irrespective of the site of activation, the subsequent ejection of the activating reagent would generate an extended iminium ion whose capture by adventitious water would produce legonmycin A directly. For context, the treatment of enol esters with oxidizing agents to give α-hydroxyketones, or halogens to give α-haloketones, is well known [36–37]. The halogen-mediated oxidation of 3-acyloxypyrroles and related compounds, to give α-hydroxy-3-ketopyrroles, is not reported but oxidations that represent equivalent reactivity have been reported [38–41].

**Scheme 3:**
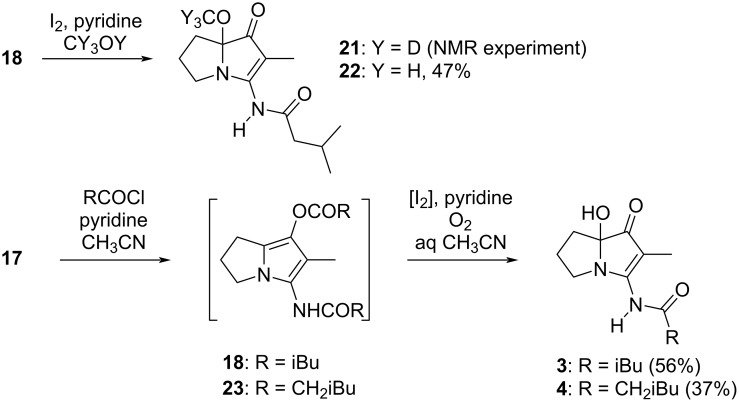
Preparation of C(7a)-functionalized pyrrolizinone derivatives and synthesis of legonmycins A and B.

The concept was tested in an NMR experiment in which a −78 °C solution of the crude diacylated species **18** in methanol-*d*_4_ was treated sequentially with a solution of I_2_ (1.0 equiv) in methanol-*d*_4_ and pyridine-*d*_5_ (≈4.5 equiv). The ^1^H NMR spectrum acquired after 20 min showed loss of the triplet at 3.76 ppm arising from the enantiotopic CH_2_N protons in pyrrole **18**, and the appearance of new resonances at 3.21 (ddd, *J* = 11.5, 9.0, 2.5 Hz, 1H) and 3.64 (dt, *J* = 11.5, 8.5 Hz, 1H) ppm that correspond visually with those reported for the diastereotopic CH_2_N protons in legonmycin A. HRMS (ESI^+^) analysis of material isolated from this NMR experiment showed *m/z* = 270.1892, suggesting that in methanol-*d*_4_ the reported [22] spectrum is not that of legonmycin A itself but that of the C(7a)-OCD_3_ derivative (**21**, Scheme 3; *m/z* calcd for C_14_H_20_D_3_N_2_O [M + H]^+^, 270.1892). A sample of the corresponding OCH_3_ derivative **22**, containing a small amount of residual toluene from the chromatography solvent, was obtained by an analogous reaction in (non-deuterated) methanol. The ^13^C NMR spectrum for this derivative corresponded closely to that obtained by us and reported for legonmycin A in methanol-*d*_4_, with an additional resonance at 51.7 ppm for the OCH_3_ carbon (3.12 ppm in the ^1^H NMR spectrum) and a shift in the C(7a) resonance.

The complication of solvent incorporation was avoided by running both the acylation and oxidative hydrolysis steps in acetonitrile, with water added in the second step. The oxidation step could be carried out either with a stoichiometric amount of I_2_, or just 10 mol % when O_2_ was bubbled gently through the reaction mixture. Additionally, since both the optimized diacylation and oxidative hydrolysis steps were carried out in acetonitrile, the transformation of the intermediate **17** into legonmycin A was conveniently performed as a one-flask process, delivering the natural product in 56% yield (corrected for the presence of ≈10 wt % toluene) on a ≈2.0 mmol scale. The repetition of the process, using isocaproyl chloride instead of isovaleryl chloride, afforded legonmycin B in moderate yield from ≈0.5 mmol of intermediate **17**. In a larger-scale reaction (≈1.1 mmol), carried out with no added I_2_, an approximately 50:50 ratio of the diacylated intermediate **23** and legonmycin B was obtained.

## Conclusion

This work demonstrates that, under carefully chosen conditions, the diacylation of pyrrolizinone derivatives such as compound **17** can be combined with the electrophilic activation and hydrolysis of the resulting electron-rich pyrroles in an overall *N-*acylation/C(7a) hydroxylation. This transformation is central to a synthesis of legonmycins A and B that requires just three laboratory operations from commercially available proline derivative **15**.

It is noteworthy that Snider’s attempt to oxidize, by epoxidation, the *N*,*O*-diacetyl derivative of compound **14** was not successful. Indeed, in our own work, the use of either NBS, MCPBA, or O_2_ with a transition-metal catalyst also gave unpromising results in attempts to oxidize compound **17**. Future work will explore whether or not the nature of the C(2)-substituent (CO_2_*t*-Bu in **15**; Me in **17**) has a significant influence on the oxidation chemistry, and how effectively these results can be translated to syntheses of further C(7a)-hydroxylated bacteral pyrrolizidines and their analogs.

## Experimental

### General experimental details

Dry solvents were obtained from an MBraun SPS-800 solvent purification system, except that THF was freshly distilled from Na/benzophenone. All other reagents or solvents were used as received from commercial suppliers, unless indicated otherwise. Column chromatography was performed using Merck Geduran Si 60 silica gel as the stationary phase unless otherwise specified. “Brine” refers to sat. aq NaCl solution. Thin-layer chromatography (TLC) was performed with Merck TLC 60 Silica gel F_254_ plates as the stationary phase and spots were visualized with KMnO_4_, vanillin, anisaldehyde, or ultraviolet light (254 nm). Retention factors (*R*_f_) are quoted to the nearest 0.05. NMR spectra were recorded on Bruker AVIII HD 500, AVII 500, or AVIII HD 400 spectrometers, 500/125 MHz spectra being recorded by the NMR Service at the Chemistry Research Laboratory, University of Oxford. Chemical shifts are reported in ppm downfield of tetramethysilane, internally referenced (in MestReNova) to the appropriate solvent peak: CDCl_3_, 7.26/77.16; DMSO-*d*_6_, 2.50/39.52; CD_3_OD, 3.31/49.00. Coupling constants (*J*) are rounded to the nearest 0.5 Hz. IR spectra were recorded on a Bruker Tensor 27 FT-IR spectrometer on a diamond ATR module, and ν_max_ values are expressed in cm^−1^. HRMS measurements were performed by the Mass Spectrometry Service at the Chemistry Research Laboratory, University of Oxford, using a Bruker microTOF spectrometer. Values of *m*/*z* are calculated to 0.0001 Daltons from the chemical formula, and all reported values are within 5 ppm of the calculated theoretical values. Melting points were recorded on a Griffin mp apparatus and are uncorrected.

#### (*S*)-*tert*-Butyl 2-(2-cyanopropanoyl)pyrrolidine-1-carboxylate (**16**)

To a solution of LHMDS (100 mL, 1.0 M in THF, 100 mmol), cooled to −78 °C and under a N_2_ atmosphere, was added dropwise propionitrile (7.33 mL, 103 mmol). The mixture was stirred vigorously at −78 °C for 40 min, after which time a solution of proline derivative **15** (11.2 g, 48.8 mmol) in THF (60 mL) was added dropwise via syringe. The mixture was warmed to rt and stirred for 42 h. The mixture was then diluted with aq acetic acid (20 mL, H_2_O/AcOH 3:1 (v/v)), concentrated, and extracted with ethyl acetate (3 × 100 mL). The combined organic extracts were washed sequentially with water (40 mL) and brine (10 mL), then dried (Na_2_SO_4_) and concentrated to afford a pale yellow solid which could be used in the next step without further purification (14.1 g, containing ≈5% w/w propionitrile and ≈10% w/w trimethylsilyl acetate). An analytical sample, a white solid, was obtained by column chromatography (pentane → pentane/ethyl acetate 4:1) as a 1:1 mixture of diastereomers, each an ≈2:1 mixture of rotamers (in CDCl_3_). *R*_f_ 0.50 (pentane/ethyl acetate 1:1); mp 92–102 °C; IR ν_max_: 2244m, 1738m, 1688s, 1394s, 1163s, 1119m; ^1^H NMR (400 MHz, CDCl_3_) δ 1.40 (s, ≈1.5H), 1.41 (s, ≈1.5H), 1.43 (s, ≈3H), 1.47 (s, ≈3H), 1.49 (d, *J* = 7.0 Hz, ≈2H), 1.51 (2 × d, *J* = 7.0 Hz, 2 × ≈0.5H), 1.83–2.43 (m, 4H), 3.39–3.62 (m, ≈2.17H), 3.63 (q, *J* = 7.0 Hz, ≈0.17H), 3.79 (2 × q, *J* = 7.0 Hz, 2 × ≈0.33H), 4.47 (dd, *J* = 8.5, 5.5 Hz, ≈0.33H), 4.55–4.60 (m, ≈0.33H), 4.62 (dd, *J* = 8.0, 5.0 Hz, ≈0.33H); ^13^C NMR (100 MHz, CDCl_3_) δ 13.5, 14.2, 14.3, 23.9, 24.8, 24.9, 28.5 (two peaks), 29.7, 29.9, 30.7, 31.0, 33.3, 33.8, 35.2, 36.2, 47.0 (three peaks), 63.3, 63.8, 64.5, 64.7, 80.5, 80.9, 81.0, 81.1, 117.8, 118.0, 118.1, 154.7, 155.4, 200.6 (two peaks), 201.0, 201.6; HRMS–ESI^+^ (*m*/*z*): [M + Na]^+^ calcd for C_13_H_20_N_2_O_3_Na, 275.1366; found, 275.1366.

#### (*S*)-3-Amino-2-methyl-5,6,7,7a-tetrahydro-1*H*-pyrrolizin-1-one hydrochloride (**17**)

Acetyl chloride (300 μL, 4.22 mmol) was added dropwise to ethanol (2 mL) stirring at −78 °C under N_2_. The mixture was briefly warmed to rt (5 min) then re-cooled to −78 °C. A solution of crude cyanoketone **16** (1.0 g, 85% purity, 3.37 mmol) in ethanol (1 mL) was added, the cooling bath replaced with a rt water bath, and stirring continued for 3.5 h. The mixture was concentrated, with azeotropic removal of ethanol and other volatiles with toluene, to afford the title compound as a caramel-colored solid (561 mg) that was used directly in the subsequent acylation and oxidation steps [the NMR spectra reproduced on page S3 of Supporting Information File 1 are for the crude material]. IR ν_max_: 3283br, 3140br, 1661m, 1607s, 1522s, 1483s, 1446s, 1382s; ^1^H NMR (400 MHz, CD_3_OD) δ 1.35–1.45 (m, 1H), 1.76 (d, *J* = 0.5 Hz, 3H), 2.23–2.32 (m, 1H), 2.32–2.46 (m, 2H), 3.24 (td, *J* = 10.0, 7.5 Hz, 1H), 3.49 (ddd, *J* = 10.0, 8.5, 2.5 Hz, 1H), 4.44 (dd, *J* = 10.5, 5.5 Hz, 1H); ^13^C NMR (100 MHz, CD_3_OD) δ 5.8, 28.4, 30.2, 46.5, 70.0, 101.3 (from a separate spectrum of a stronger but impure sample), 174.4, 176.7; HRMS–ESI^+^ (*m*/*z*): [M]^+^ calcd for C_8_H_13_N_2_O, 153.1022; found, 153.1022. The free-base was also prepared in a separate large-scale experiment [from crude **16** (17.6 g, 69.8 mmol) giving hydrochloride **17** (13.1 g, 69.4 mmol)]. Thus, a 6.4 g sample of the crude salt **17** was dissolved in methanol/triethylamine 9:1, concentrated, then re-dissolved in the minimum amount of methanol to allow precipitation of triethylamine hydrochloride by the addition of THF. The solid was filtered off and the filtrate concentrated. The residue was purified by two sequential rounds of column chromatography (ethyl acetate/triethylamine 99:1 → ethyl acetate/methanol/triethylamine 89:10:1) to produce the pyrrolizinone free-base (2.28 g, 15.0 mmol, equating to 44% overall yield from proline derivative **16**). *R*_f_ 0.10 (ethyl acetate/methanol 9:1); ^1^H NMR (400 MHz, CDCl_3_) δ [the resonances vary with sample concentration and source of CDCl_3_] 1.56–1.65 (m, 1H) overlaying 1.60 (s, 3H), 1.94–2.04 (m, 2H), 2.10–2.29 (m, 1H), 3.08–3.15 (m, 1H), 3.17–3.23 (m, 1H), 3.86 (t, *J* = 8.0 Hz, 1H), 4.67 (br s, 2H); ^13^C NMR (100 MHz, CD_3_OD) δ 5.6, 28.1, 29.0, 47.9, 71.1, 93.0, 177.6, 194.3.

#### *N*-(7a-Methoxy-2-methyl-1-oxo-5,6,7,7a-tetrahydro-1*H*-pyrrolizin-3-yl)-3-methylbutanamide, *O-*methyl-legonmycin A (**22**)

*This experiment was performed as described in order to trial various work-up and chromatography options.* To a mixture of 3-aminopyrrolizine hydrochloride derivative **17** (2.0 g, 10.6 mmol), distilled pyridine (3.42 mL, 42.4 mmol), and acetonitrile (21 mL) at rt was added isovaleryl chloride (2.58 mL, 21.2 mmol). The mixture was stirred for 1 h, then half of the reaction mixture was removed via syringe to a second flask and concentrated (the remaining half was used for a separate reaction). The residue was dissolved in methanol (53 mL) then the stirred mixture was cooled to −78 °C and a pre-cooled (−78 °C) solution of I_2_ (1.35 g, 5.32 mmol) in methanol (53 mL) was added via cannula over 15 min. The reaction mixture was allowed to warm to rt over 14 h, the volatiles were removed, then pyridine (2 mL) and activated charcoal (5 g) were added. The resulting slurry was part-purified by column chromatography (dichloromethane → methanol) and the product-containing fractions were combined and recolumned (dichloromethane → dichloromethane/methanol 9:1). The product-containing fractions were combined and diluted with CuSO_4_·5H_2_O solution (20 mL; saturated, in methanol), shaken with activated carbon (5 g), filtered, and concentrated to afford a brown oil (1.33 g). This residue was subjected to a third round of chromatography (toluene/ethyl acetate 3:2 → toluene/ethyl acetate/acetone 2:2:1) to afford the title compound as an oil (661 mg, 47%). *R*_f_ 0.35 (dichloromethane/methanol 9:1); ^1^H NMR (400 MHz, CD_3_OD) δ 1.02 (d, *J* = 6.5 Hz, 3H), 1.03 (d, *J* = 6.5 Hz, 3H), 1.59 (s, 3H), 1.64 (ddd, *J* = 13.0, 10.5, 7.5 Hz, 1H), 1.94 (ddd, *J* = 13.0, 6.5, 3.0 Hz, 1H), 1.98–2.09 (m, 1H), 2.16 (≈ nonet, apparent *J* = 7.0 Hz, 1H), 2.32–2.34 (m, partially obscured by residual PhC*H*_3_, 1H), 2.34 (dd, *J* = 14.5, 7.0 Hz, 1H), 2.40 (dd, *J =* 14.5, 7.0 Hz, 1H), 3.10 (≈dt, *J* = 11.0, 8.0 Hz, 1H) overlays 3.12 (s, 3H), 3.37 (ddd, *J* = 11.0, 8.0, 3.5 Hz, 1H); ^13^C NMR (100 MHz, CD_3_OD) δ 6.7, 22.6, 22.7, 27.2, 27.7, 33.4, 46.6, 49.3, 51.7, 101.6, 102.0, 173.1, 199.9 (one resonance not resolved); LRMS–ESI^+^ (*m*/*z*): 289.1 ([M + Na]^+^, 26%), 235.2 ([M + H – MeOH]^+^, 100%), 151.0 (24%); HRMS–ESI^+^ (*m*/*z*): [M − OMe]^+^ calcd for C_13_H_19_N_2_O_2_, 235.1441; found, 235.1443.

#### *N*-(7a-Hydroxy-2-methyl-1-oxo-5,6,7,7a-tetrahydro-1*H*-pyrrolizin-3-yl)-3-methylbutanamide, legonmycin A (**3**)

To a mixture of 3-aminopyrrolizine hydrochloride derivative **17** (412 mg, 2.18 mmol), distilled pyridine (882 µL, 10.9 mmol), and acetonitrile (4.36 mL) at rt was added isovaleryl chloride (532 µL, 4.36 mmol). The mixture was stirred for 1 h, then a solution of I_2_ (54.5 mg, 0.215 mmol) in water (2.2 mL) and acetonitrile (15.2 mL) was added. O_2_ was bubbled gently through the solution for 15 h, and the mixture was concentrated with azeotropic removal of water with toluene (2 × 50 mL). The residue was purified by column chromatography (toluene/ethyl acetate/acetone 3:2:0–12:8:5) to afford the title compound as a yellow amorphous solid (345 mg, 56% corrected for the presence of ≈10 wt % toluene). The data are consistent with literature values [22]. *R*_f_ 0.10 (toluene/ethyl acetate/acetone 2:2:1); mp 128 °C [lit. mp not given]; IR ν_max_ 3234br, 2958m, 1667m, 1571s, 1517s, 1437s, 1197m, 730m; ^1^H NMR (400 MHz, CD_3_OD) δ 1.01 (d, *J* = 6.5 Hz, 3H), 1.02 (d, *J* = 6.5 Hz, 3H), 1.58 (s, 3H), 1.65 (ddd, *J* = 13.0, 10.5, 7.5 Hz, 1H), 1.93 (ddd, *J* = 13.0, 6.5, 3.5 Hz, 1H), 1.99–2.11 (m, 1H), 2.15 (nonet, apparent *J* = 6.5 Hz, 1H), 2.32 (dd, *J* = 14.5, 7.0 Hz, 1H) overlaying 2.28–2.39 (m, 1H), 2.38 (dd, *J* = 14.5, 7.0 Hz, 1H), 3.04 (ddd, *J* = 11.0, 8.5, 7.0 Hz, 1H), 3.43 (ddd, *J* = 11.0, 8.0, 4.0 Hz, 1H); ^13^C NMR (100 MHz, CD_3_OD) δ 7.0, 22.6, 22.7, 27.2, 27.8, 33.8, 46.6, 49.3, 97.7, 99.4, 169.4, 172.9 (carbonyl resonance at ≈201–202 not seen); HRMS–ESI^+^ (*m*/*z*): [M – OH]^+^ calcd for C_13_H_19_N_2_O_2_, 235.1441; found, 235.1439.

#### *N*-(7a-Hydroxy-2-methyl-1-oxo-5,6,7,7a-tetrahydro-1*H*-pyrrolizin-3-yl)-3-methylpentanamide, legonmycin B (**4**)

Two separate small-scale diacylation reactions were run in parallel and then combined for oxidative hydrolysis. The quantities for each reaction are separated by a forward slash. To a mixture of 3-aminopyrrolizine hydrochloride **17** (22.3/43.6 mg, 0.118/0.231 mmol) and a solution of distilled pyridine in acetonitrile (1.20/2.30 mL, 0.5 M, 0.590/1.15 mmol) was added isocaproyl chloride (32/63 µL, 0.237/0.468 mmol). Both reaction mixtures were stirred for 18 h and then combined. Water (350 µL) and I_2_ (8.4 mg, 0.0331 mmol) were added sequentially. O_2_ was bubbled gently through the solution for 21 h and the mixture was concentrated. The residue was purified by column chromatography (toluene/ethyl acetate/acetone 3:2:0–0:1:1) to afford the title compound as a yellow glass (34.7 mg, 37%). The data are consistent with literature values [22]. *R*_f_ 0.40 (acetone/ethyl acetate/toluene 2:1:1); mp 49 °C [lit. mp not given]; IR ν_max_ 3234br, 2957m, 1698m, 1668m, 1571s, 1454m, 1195m; ^1^H NMR (400 MHz, DMSO-*d*_6_) δ 0.88 (d, *J* = 6.5 Hz, 3H), 0.89 (d, *J* = 6.5 Hz, 3H), 1.42–1.60 (m, 4H) overlaying 1.44 (s, 3H), 1.71 (ddd, *J* = 13.0, 6.5, 4.0 Hz, 1H), 1.77–1.87 (m, 1H), 2.05–2.17 (m, 1H), 2.37 (dd, *J* = 15.0, 7.5 Hz, 1H), 2.44 (dd, *J* = 15.0, 7.5 Hz, 1H), 2.91 (dt, *J* = 11.0, 7.5 Hz, 1H), 3.20 (ddd, *J* = 11.0, 7.5, 4.5 Hz, 1H), 6.04 (s, 1H), 10.16 (s, 1H); ^13^C NMR (100 MHz, DMSO-*d*_6_) δ 7.3, 22.2 (two peaks), 26.1, 27.2, 32.9, 33.7, 34.0, 47.6, 95.6, 97.3, 166.1, 170.9, 198.8; HRMS–ESI^+^ (*m*/*z*): [M + H]^+^ calcd for C_14_H_23_N_2_O_3_, 267.1703; found, 267.1703.

## Supporting Information

File 1 Copies of the NMR spectra for compounds **16**, **17** (crude HCl salt and purified free-base), **22**, **3** (legonmycin A), and **4** (legonmycin B).
