# Supplementary material for: Synthesis of legonmycins A and B, C(7a)-hydroxylated bacterial pyrrolizidines
Source: Beilstein J Org Chem. 2021 Feb 2;17:334–42. doi: 10.3762/bjoc.17.31 (PMC7871033; doi:10.3762/bjoc.17.31)

BEILSTEIN JOURNAL OF ORGANIC CHEMISTRY

Supporting Information

for

Synthesis of legonmycins A and B, C(7a)-hydroxylated bacterial pyrrolizidines

Wilfred J. M. Lewis, David M. Shaw and Jeremy Robertson

Beilstein J. Org. Chem. **2021,** *17,* 334–342. doi:10.3762/bjoc.17.31

Copies of the NMR spectra for compounds 16, 17 (crude HCl salt and purified free-base), 22, 3 (legonmycin A), and 4 (legonmycin B)

License and Terms: This is a supporting information file under the terms of the Creative Commons Attribution License (https://creativecommons.org/licenses/by/4.0). Please note that the reuse, redistribution and reproduction in particular requires that the author(s) and source are credited and that individual graphics may be subject to special legal provisions.
The license is subject to the *Beilstein Journal of Organic Chemistry* terms and conditions: (https://www.beilstein-journals.org/bjoc/terms)

16 ^{1}H NMR (400 MHz, $CDCl_3$) dr ~1:1, each diastereomer ~2:1 ratio of *N*-Boc rotamers

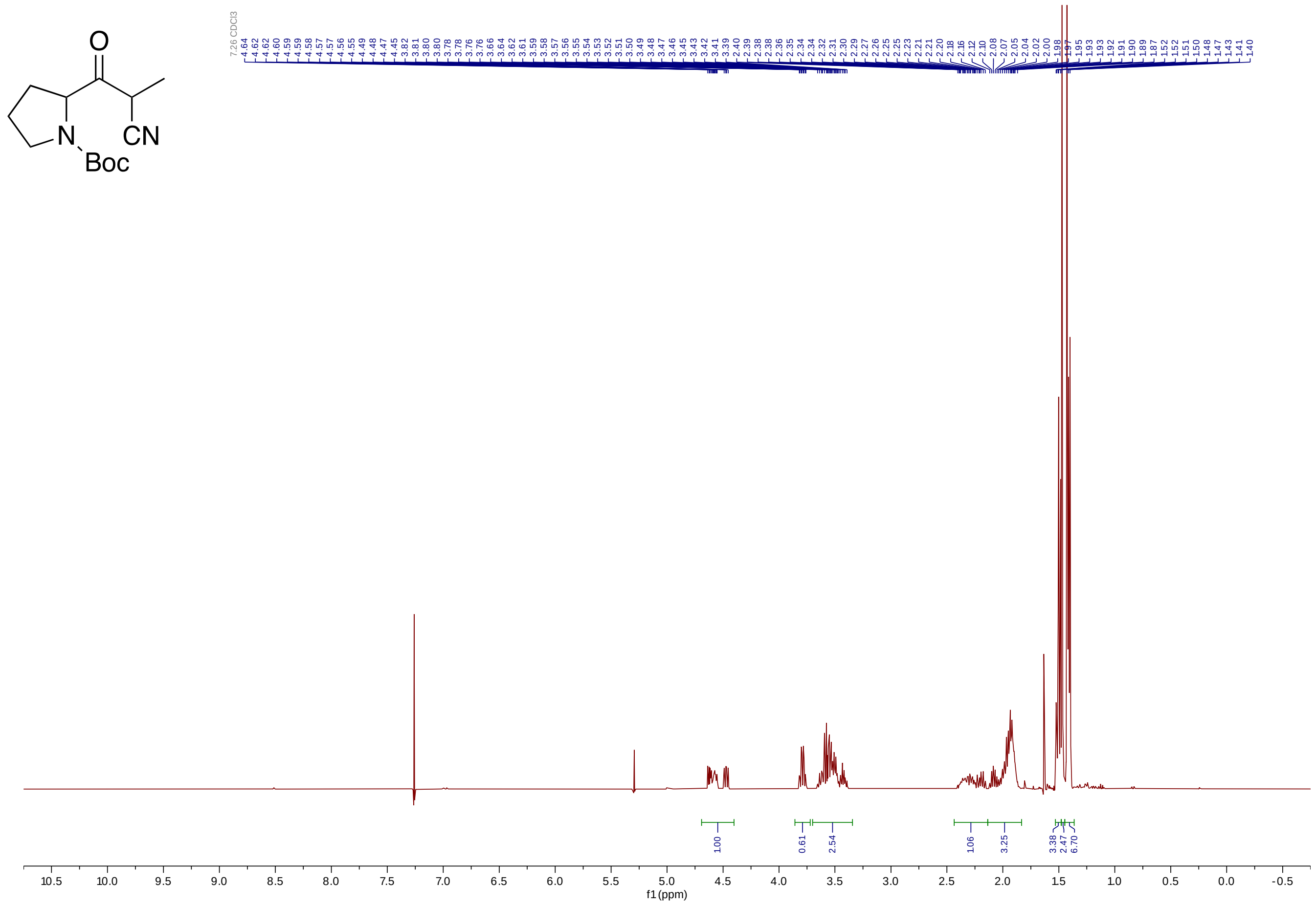

16 ^{13}C NMR (100 MHz, $CDCl_3$) dr ~1:1, each diastereomer ~2:1 ratio of *N*-Boc rotamers

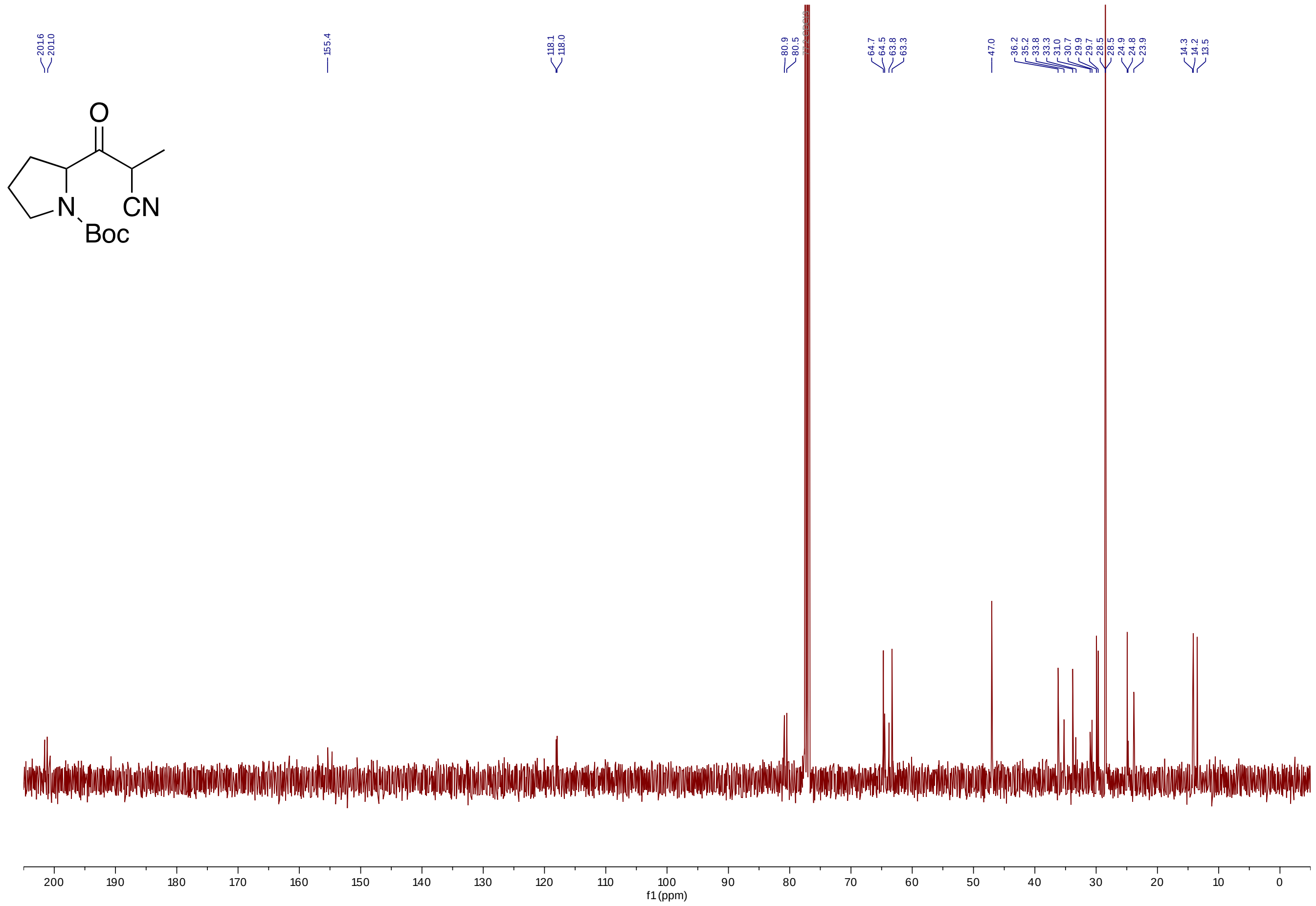

17 ^{1}H NMR (400 MHz, CD_3OD) crude, HCl salt

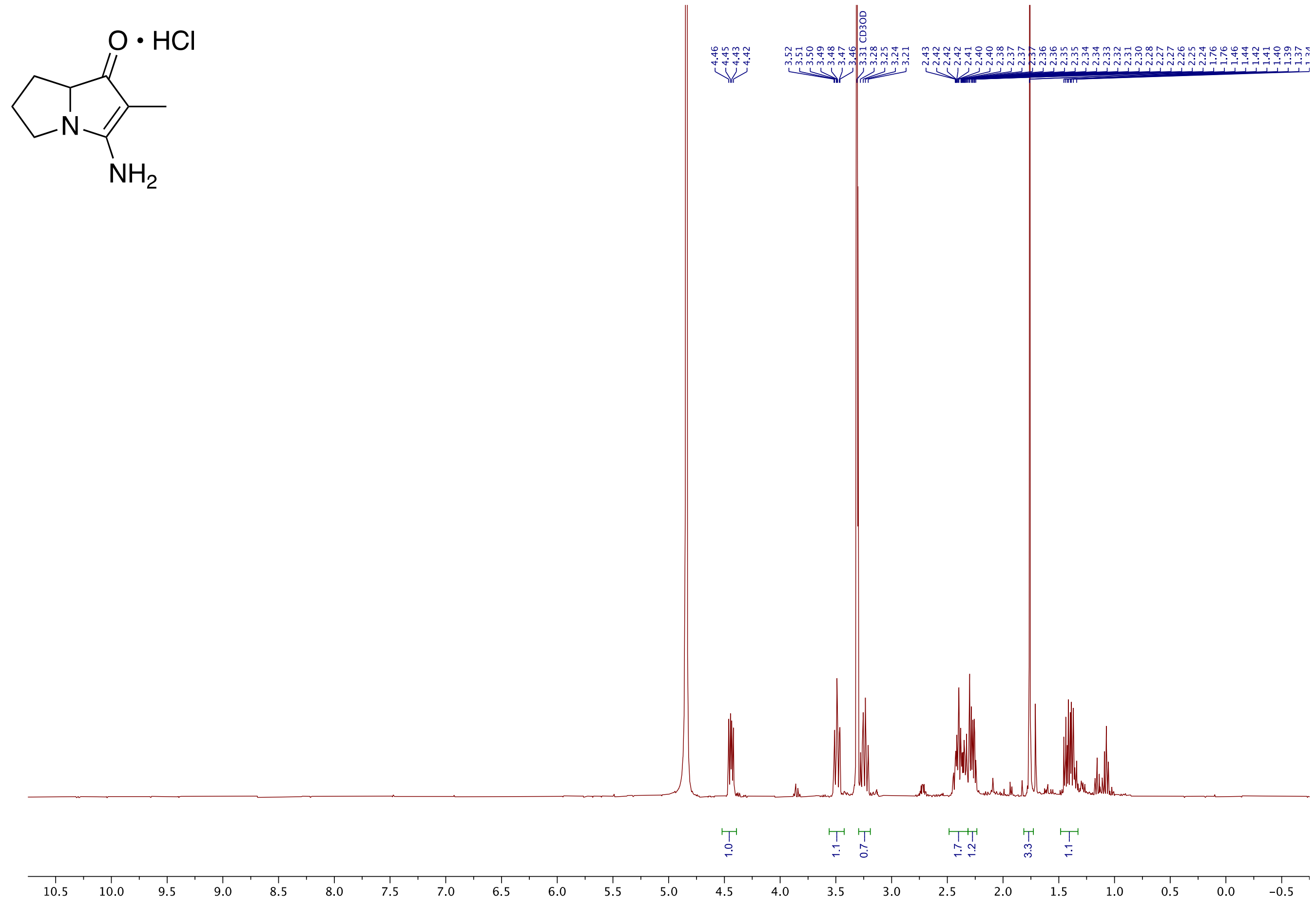

17 ^{13}C NMR (100 MHz, CD_3OD) crude, HCl salt

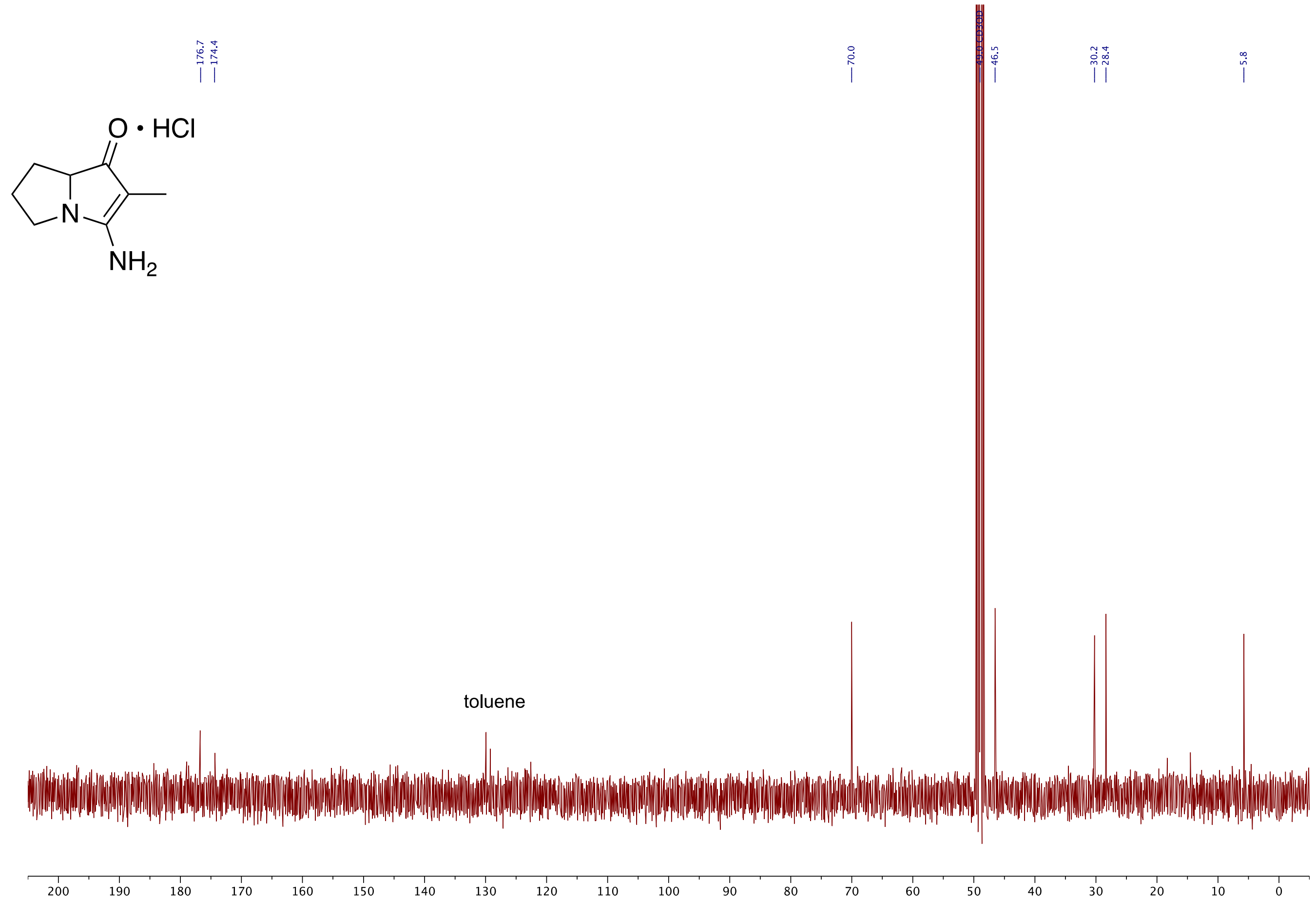

17 ^{1}H NMR (400 MHz, $CDCl_3$) freebase, after chromatography

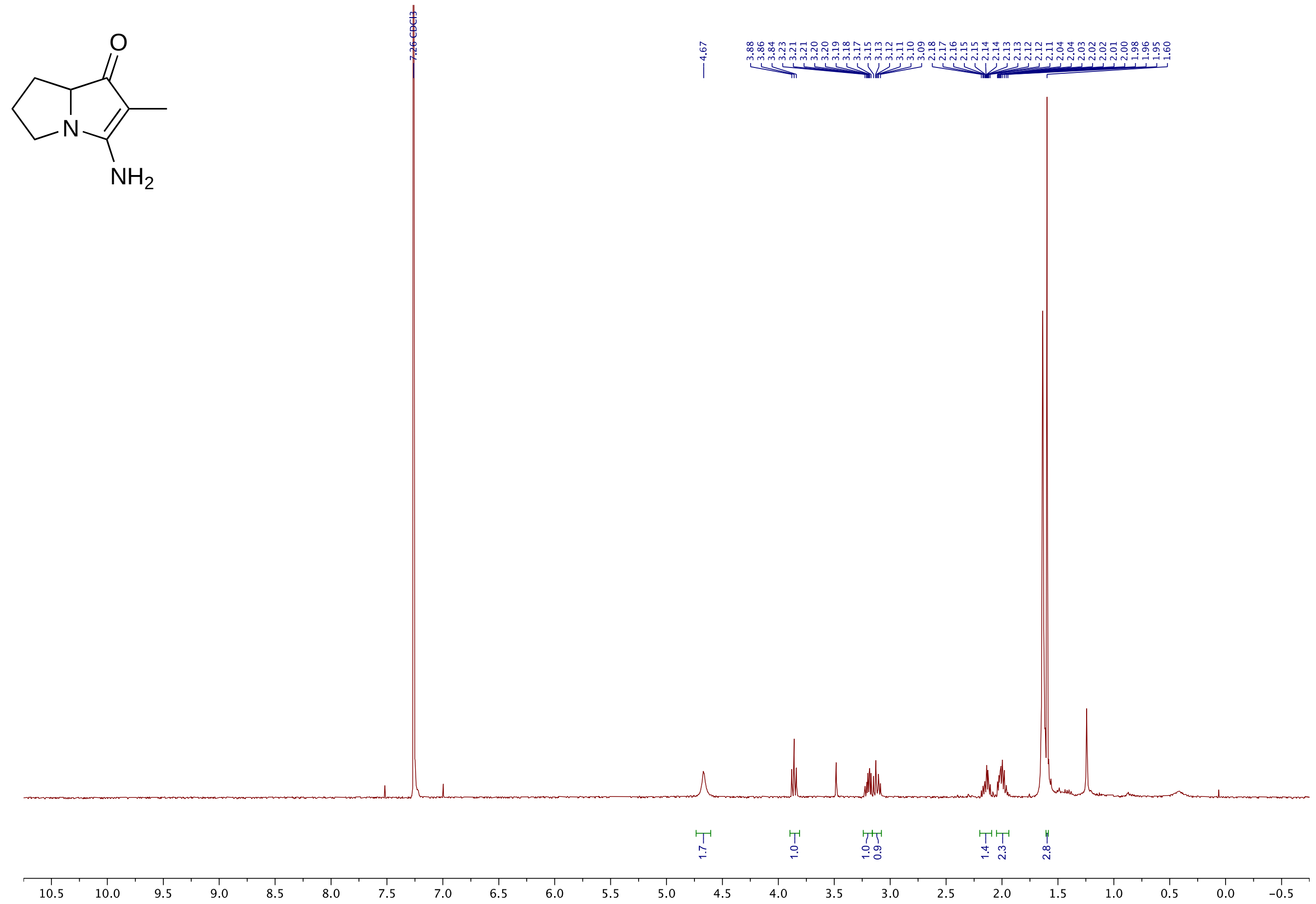

17 ^{13}C NMR (100 MHz, CD_3OD) freebase, after chromatography

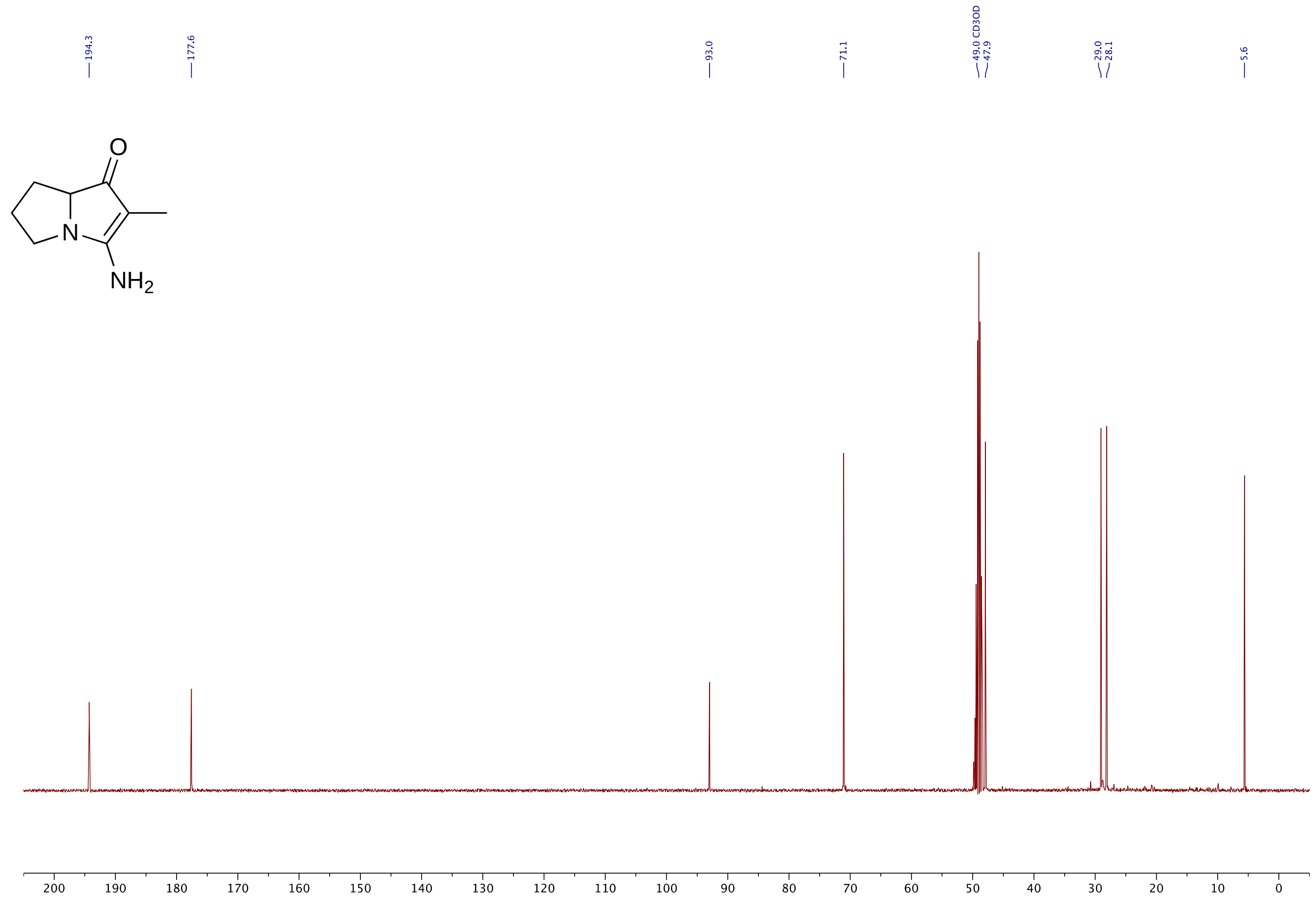

22 ^{1}H NMR (400 MHz, CD_3OD)

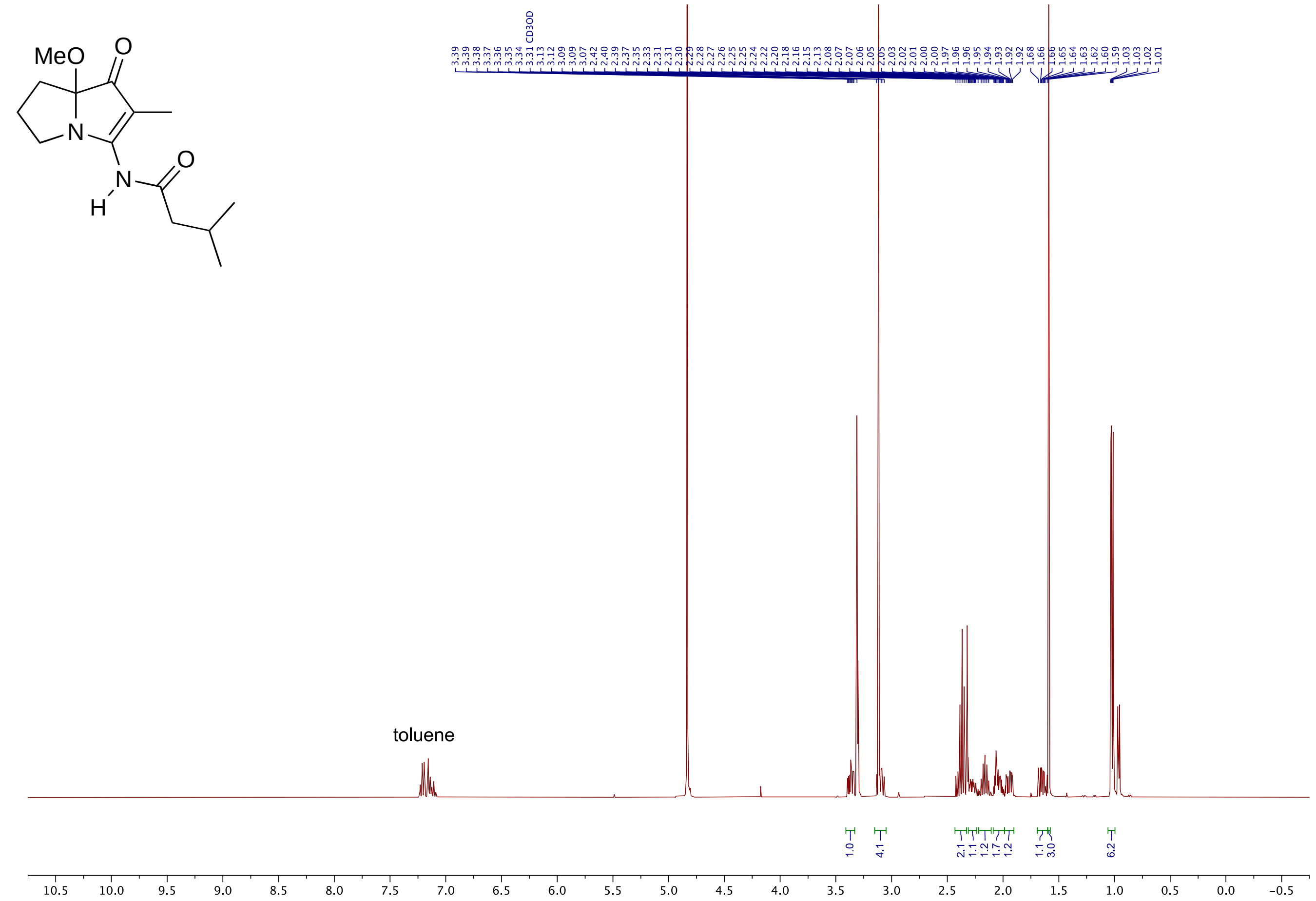

22 ^{13}C NMR, 100 MHz, CD_3OD)

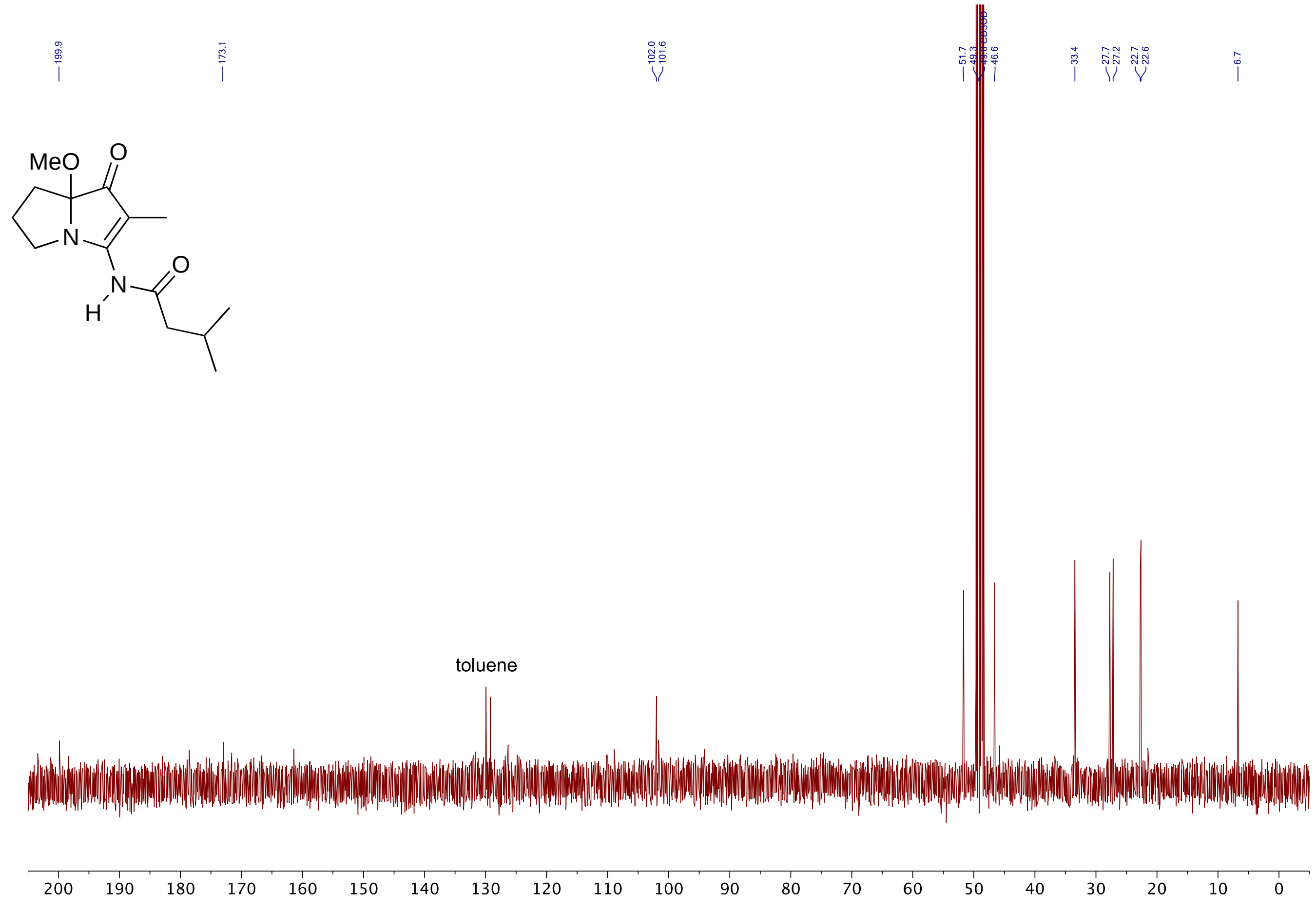

3 ^{1}H NMR (400 MHz, CD_3OD)

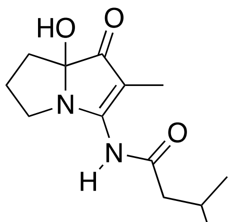

legonmycin A

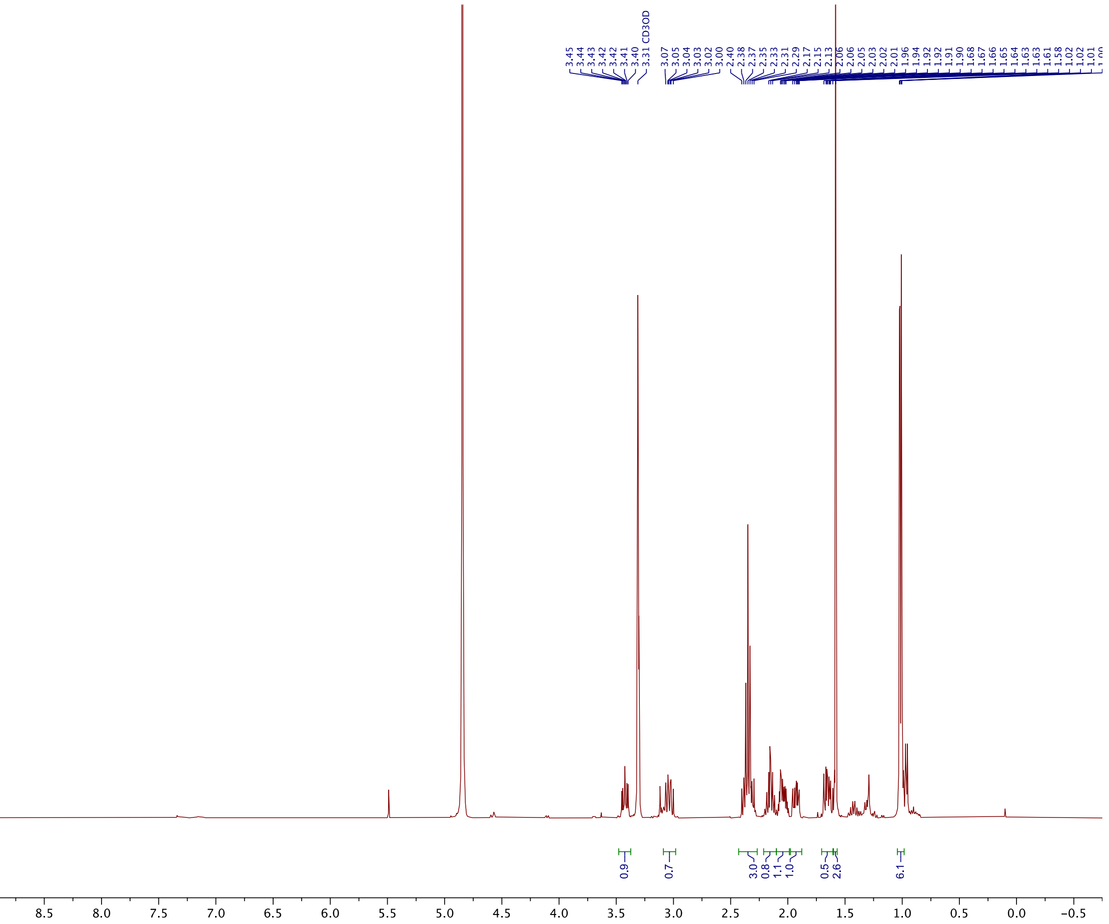

3 ^{13}C NMR (100 MHz, CD_3OD) contains ~10 wt% toluene

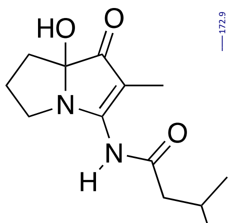

legonmycin A

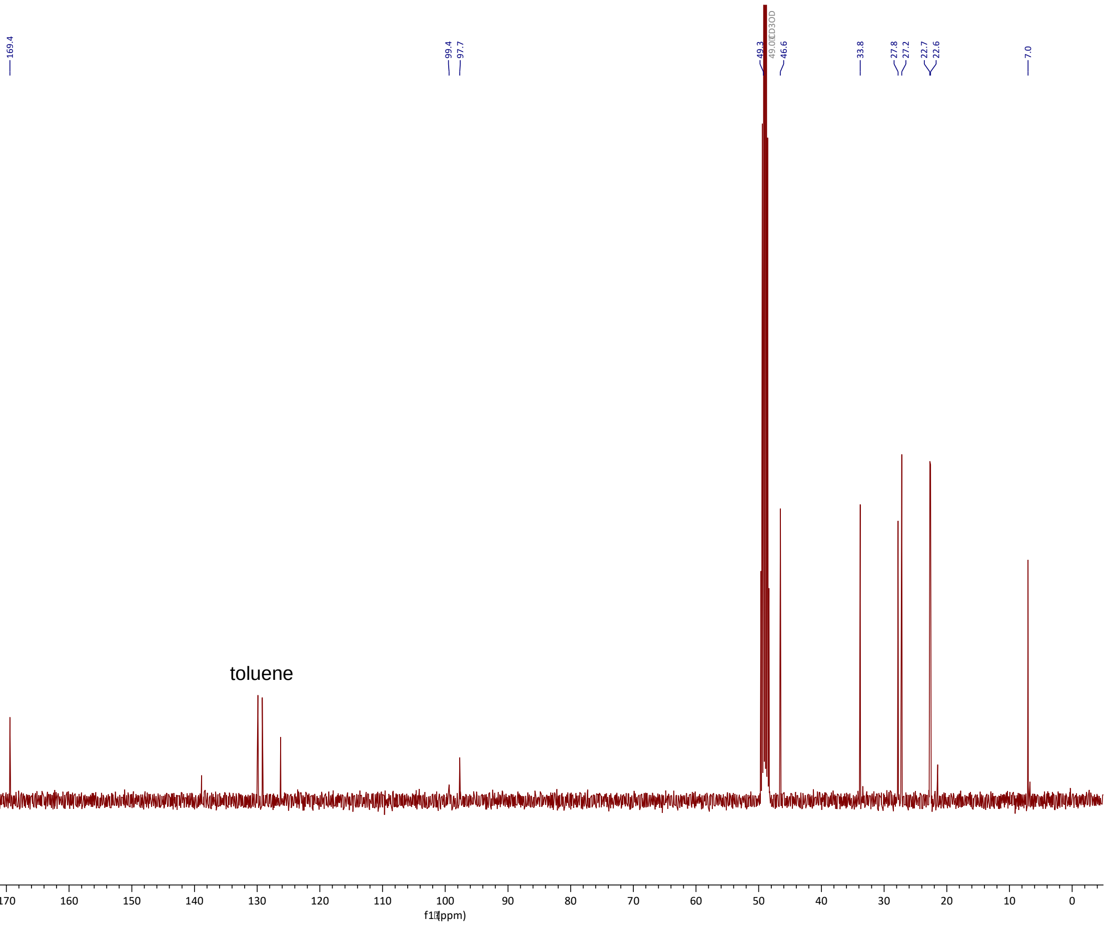

4 ^{1}H NMR (400 MHz, DMSO-d_6)

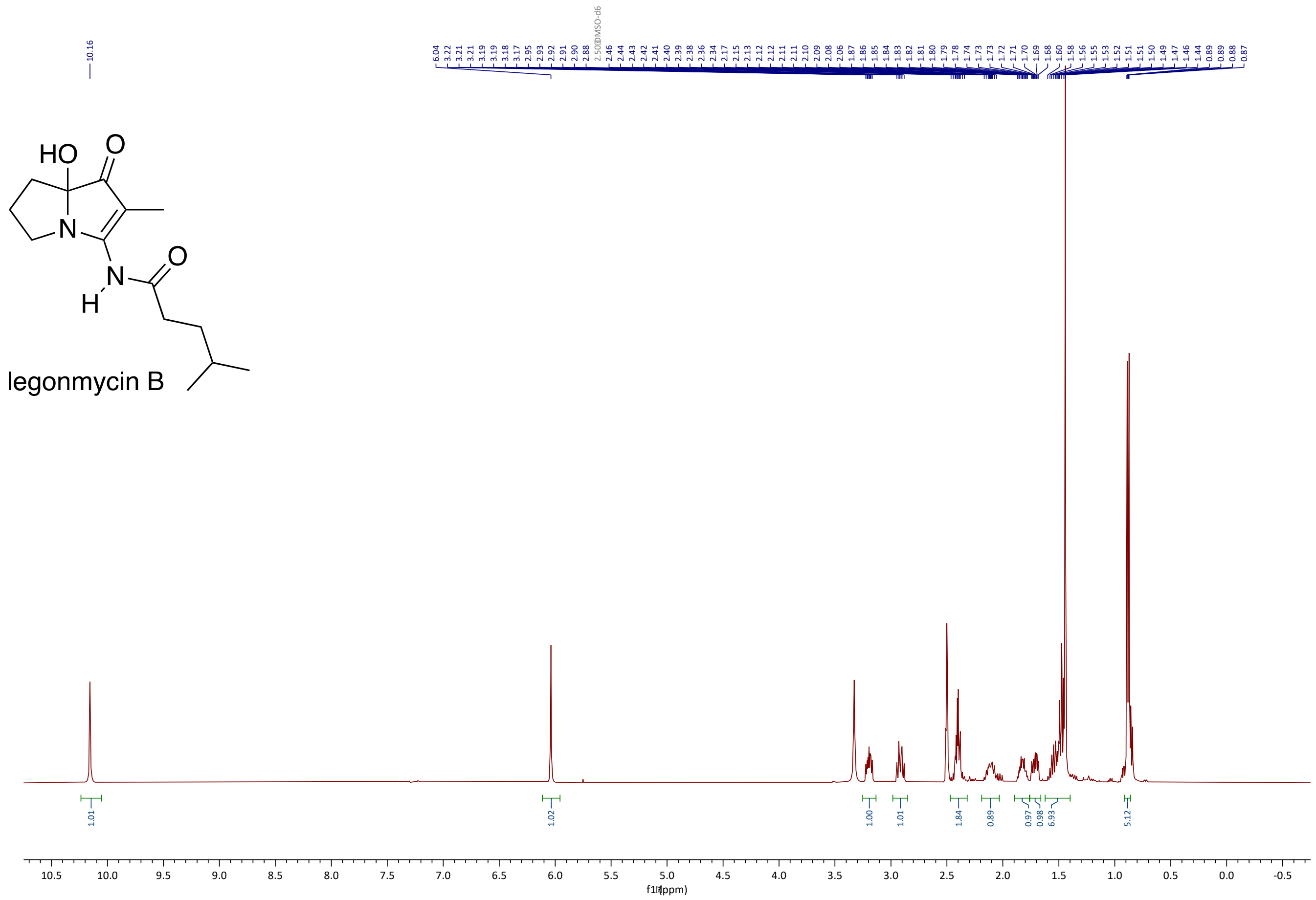

4 ^{13}C NMR (100 MHz, DMSO-d_6)

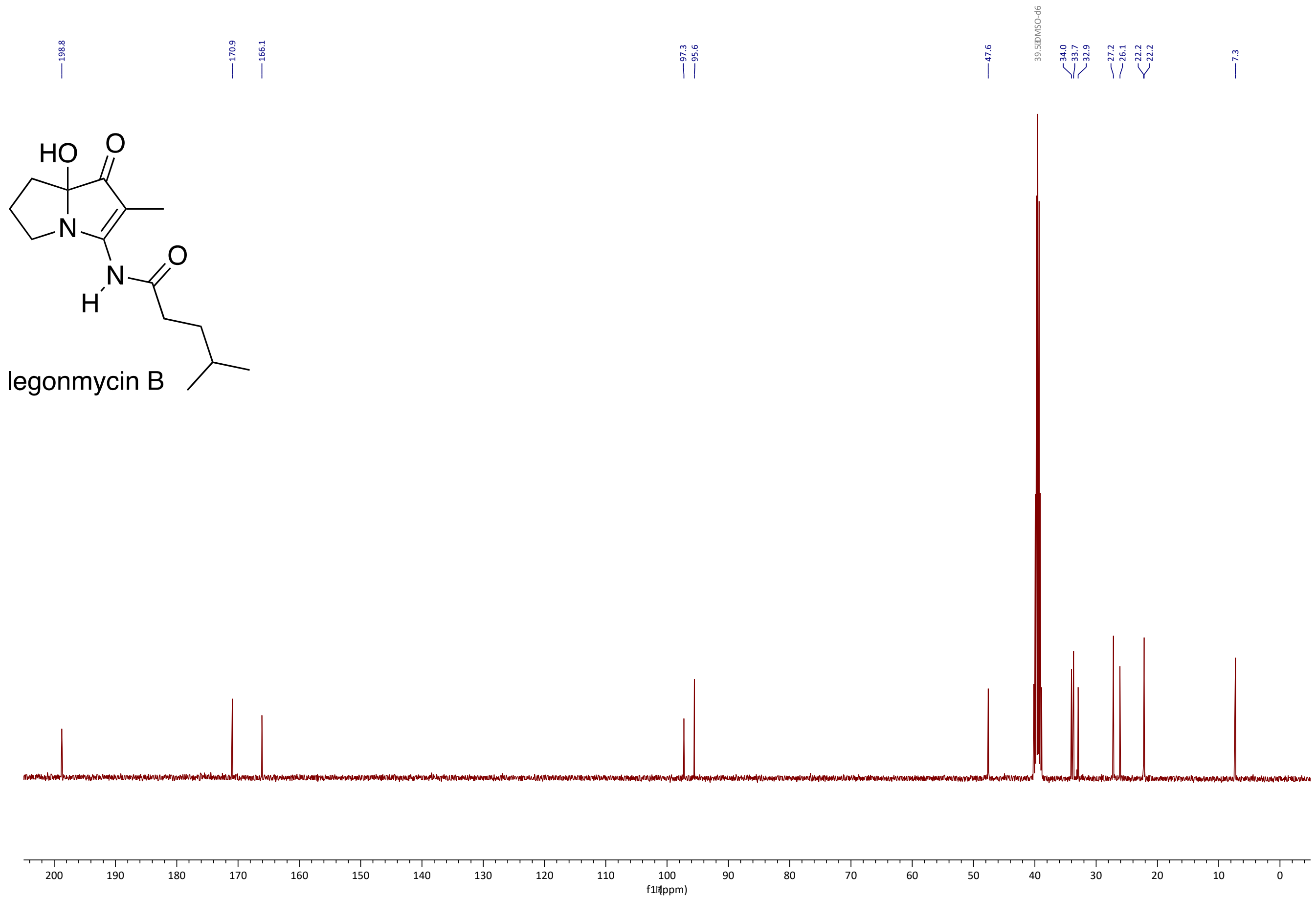

Supplement: File 1 — Copies of the NMR spectra for compounds 16, 17 (crude HCl salt and purified free-base), 22, 3 (legonmycin A), and 4 (legonmycin B). [file Beilstein_J_Org_Chem-17-334-s001.pdf]
